# New discoveries of *Lanonia* (Arecaceae) from northern Vietnam and southwestern China

**DOI:** 10.3897/phytokeys.279.207204

**Published:** 2026-09-17

**Authors:** Jia-Wei Qin, Zhi-Ling Tian, Zheng-Shun Chen, Khang Sinh Nguyen, Ren-Ping Kuang

**Affiliations:** 1 Hunan Research Center of the Basic Discipline Developmental Biology, College of Life Sciences, Hunan Normal University, Changsha 410081, China Hunan Research Center of the Basic Discipline Developmental Biology, College of Life Sciences, Hunan Normal University Changsha China; 2 Institute of Biology (IB), Vietnam Academy of Science and Technology (VAST), 18 Hoang Quoc Viet, Cau Giay, Hanoi 10072, Vietnam Institute of Biology (IB), Vietnam Academy of Science and Technology (VAST) Hanoi Vietnam

**Keywords:** *
Lanonia
*, Palmae, phylogeny, pollen morphology, taxonomy, Vietnam, China

## Abstract

A new species of *Lanonia* (Arecaceae, Coryphoideae, Livistoninae) from Lao Cai, Vietnam, *Lanonia nhatii***sp. nov**., is described and illustrated, and a new record of *Lanonia yangchunensis* in Guangxi, China, is described based on morphological comparisons, molecular phylogenetic analysis, and pollen morphology. *L. nhatii* is distinct from all other congeners by its extremely short costa and purplish-black mature fruit. Additional morphological characters, especially variable leaf forms, were found in *L. yangchunensis*. Phylogenetic analyses using maximum likelihood (ML) and Bayesian inference (BI) methods based on multigene concatenation, including *CISP4*, *CISP5*, *MS*, *RPB2*, *matK*, *ndhF*, and *trnD-trnT* sequences, provide further support for recognition of *L. nhatii* as a distinct lineage (ML bootstrap = 100%, BI posterior probability [PP] = 1.00) and of *L. yangchunensis* with different leaf forms as a monophyletic clade (ML bootstrap = 100%, BIPP = 1.00). Pollen morphology of these two species was investigated and described using scanning electron microscopy (SEM).

## Introduction

The genus *Lanonia* A.J.Hend. & C.D.Bacon belongs to the subtribe Livistoninae, tribe Trachycarpeae (Arecaceae: Coryphoideae) (Dransfield et al. 2008). A molecular phylogenetic study by Henderson and Bacon (2011) indicated that species formerly in *Licuala* Wurmb did not form a monophyletic lineage, and eight species were segregated to constitute the new genus *Lanonia*. Recent phylogenomic analyses further demonstrate that *Lanonia* and *Licuala* are distantly related (Wrisberg et al. 2025; Bellot et al. 2026). Although both genera share the diagnostic feature of wedge-shaped leaf segments with reduplicate margins, *Lanonia* can be distinguished from *Licuala* by characters including dioecy, dimorphic inflorescences, leaves with the central segment split to the apex of the costa, and the costa with an abaxial pulvinus (Henderson and Nguyễn 2022). However, exceptions were observed, including *Lanonia* species with an unsplit central segment and a continuous costa, species with monomorphic inflorescences, and specimens bearing hermaphrodite flowers (Henderson et al. 2008; Henderson and Nguyễn 2017, 2019, 2022; Chen et al. 2025). This suggests that accurate differentiation between *Lanonia* and *Licuala* relies on both morphology and molecular analysis.

Currently comprising 21 species, *Lanonia* is distributed in China, Vietnam, Laos, and Indonesia (Jin et al. 2026; POWO 2026). There are four native species in China: *Lanonia dasyantha* (Burret) A.J.Hend. & C.D.Bacon, *Lanonia hainanensis* (A.J.Hend., L.X.Guo & Barfod) A.J.Hend. & C.D.Bacon, *Lanonia yunnanensis* R.P.Kuang & J.W.Qin, and *Lanonia yangchunensis* Y.S.Chen (Henderson and Nguyễn 2022; Chen et al. 2025; Jin et al. 2026). Two *Lanonia* species were encountered during surveys of indigenous palms in the China–Vietnam border region. Based on evidence from morphological comparisons and phylogenetic analysis of closely related species, one was confirmed to be a hitherto undescribed species, and the other was confirmed as *L. yangchunensis*, a new regional record for Guangxi, China. In this study, the new species is described, and additional morphological data on *L. yangchunensis* are provided.

## Methods

### Morphological studies

The materials studied for the undescribed *Lanonia* species were collected from Lao Cai Province, Vietnam, and those of *L. yangchunensis* were collected from Guangxi Zhuang Autonomous Region, China. Taxonomic characters were selected following the genus description of *Lanonia* provided by Henderson and Nguyễn (2022). Morphological observations, measurements, and high-quality photographic recording were conducted both in the field and in the laboratory based on fresh material.

### Sampling and sequencing

Leaf tissue samples from three individuals of the undescribed species and four mature individuals of *L. yangchunensis* exhibiting different leaf forms (undivided, bifid, quadrifid, and hexafid) were collected for this study. DNA extraction was performed using the cetyltrimethylammonium bromide (CTAB) method described by Porebski et al. (1997). Seven molecular markers were selected for sequencing based on Henderson and Bacon (2011), comprising four nuclear loci (*CISP4*, *CISP5*, *MS*, and *RPB2*) and three plastid loci (*matK*, *ndhF*, and *trnD-trnT*). Primer sequences are listed in Table 1, and the PCR amplification protocols were adapted from the methods described in the cited references. PCR products were sequenced by Tsingke Biotechnology Co., Ltd. (Wuhan, Hubei, China), yielding 47 sequences.

**Table 1.** Details of primers employed herein.

**Gene**	**Primer name**	**Primer sequence (5’-3’)**	**References**
*CISP4*	ORSC2_007_F	GGAGATCAACGTCTTCTTGT	Bacon et al. 2008
	ORSC2_007_R	GCATACTCAGGAGCACAATA	Bacon et al. 2008
*CISP5*	ORSC8_001_F	CACAGAGAAGTTAAGTGCCA	Bacon et al. 2008
	ORSC8_001_R	ATCAAGGAGTACCGTGGCAA	Bacon et al. 2008
*MS*	ms-livistona1F	TTTGGGCCTTTCTTCTAT	Crisp et al. 2010
	ms-livistona1R	ATTCCGACCCTGGTGTTC	Crisp et al. 2010
*RPB2*	RPB2-P10F	CARGARGATATGCCATGGAC	Denton et al. 1998
	RPB2-M11R	CCACGCATCTGATATCCAC	Roncal et al. 2005
*matK*	matK-19F	CGTTCTGACCATATTGCACTATG	Wang 2007
	matK-1862R	CATTGCACACGACTTTACC	Asmussen et al. 2006
*ndhF*	ndhF1001-1F	ATGGAACAKACATATSAATAT	Olmstead and Sweere 1994
	ndhF2110R	CCCCCTAYATATTTGATACCTTCTCC	Olmstead and Sweere 1994
	ndhF-internal-1F	ATCTGCACAATTCCCCCTCC	Designed by the author
	ndhF-internal-1R	TTTGGTGCTTCTTTTCCCCA	Designed by the author
*trnD*-*trnT*	trnD (GUC)	ACCAATTGAACTACAATCCC	Demesure et al. 1995
	trnT (GGU)	CTACCACTGAGTTAAAAGGG	Demesure et al. 1995

### Phylogenetic analysis

To construct a phylogenetic tree of the subtribe Livistoninae (comprising six genera, i.e., *Licuala*, *Saribus* Blume, *Pholidocarpus* Blume, *Lanonia*, *Johannesteijsmannia* H.E.Moore, and *Livistona* R.Br.), a total of 438 sequences from 70 species (including the outgroup, *Serenoa repens* (W.Bartram) Small) were obtained from NCBI GenBank. The accession numbers of all nucleotide sequences used in this study (both novel sequences and those derived from GenBank) are listed in Suppl. material 1.

Phylogenetic analyses were conducted using PhyloSuite v. 1.2.3 (Zhang et al. 2020; Xiang et al. 2023) following the workflow below. Nucleotide sequences were aligned with MAFFT v. 7.505 (Katoh and Standley 2013) under default parameters. Gap sites were removed using trimAl v. 1.2rev57 (Capella-Gutierrez et al. 2009). ModelFinder v. 2.2.0 (Kalyaanamoorthy et al. 2017) was employed to select the best partitioning scheme and evolutionary models for seven predefined partitions under the Bayesian information criterion (BIC). Maximum likelihood (ML) phylogenies were inferred with IQ-TREE v. 2.2.0 (Nguyễn et al. 2015) using the TIM2+F+1+1+R3 model with 1,000 bootstrap replicates, while Bayesian inference (BI) phylogenies were inferred using MrBayes v. 3.2.7a (Ronquist et al. 2012) under the GTR+F+I+G4 model with two parallel runs of 2 × 10^6^ generations, sampling every 1,000 generations and discarding the initial 25% of trees as burn-in. Phylogenetic trees were visualized using TreeGraph2 (Stöver and Müller 2010).

### Pollen morphology

Staminate inflorescences were collected in the field. The dehydrated pollen grains were placed on a copper platform with double-sided adhesive tape and air-dried. The sample holder was sputter-coated with a 2 nm gold layer using a fully automated sputter coater (JEOL Auto Fine Coater JEC-3000FC). Pollen grains were imaged using an electron microscope (JEOL InTouchScope™ JSM-IT500) and described based on the work of Ambwani and Kumar (1993) and Punt et al. (2007).

## Results

### Taxonomic treatments

#### Lanonia nhatii

Taxon classificationPlantaeArecalesArecaceae

J.W.Qin, R.P.Kuang & K.S.Nguyen sp. nov.

FEA855A0-D027-5AD8-A99A-C5F61D97999A

urn:lsid:ipni.org:names:77396872-1

Figs 1, 2

##### Type.

Vietnam • Lao Cai Province, Chieng Ken Coummune, precise locality withheld, elevation 618 m a.s.l., growing in primary evergreen broad-leaved forest, 23 February 2026, *Jia-Wei Qin*, *QJW-VNLC 260223* (***holotype***: HNNU!); and ***paratypes***: • ibid., 20 January 2026, *Trieu Van Nhat*, *LCT-TVN 001* (HN!); • ibid., 24 February 2026, *Jia-Wei Qin*, *QJW-VNLC 260224* (HNNU! HITBC! BAZI! KUN! IBK!).

**Figure 1.**
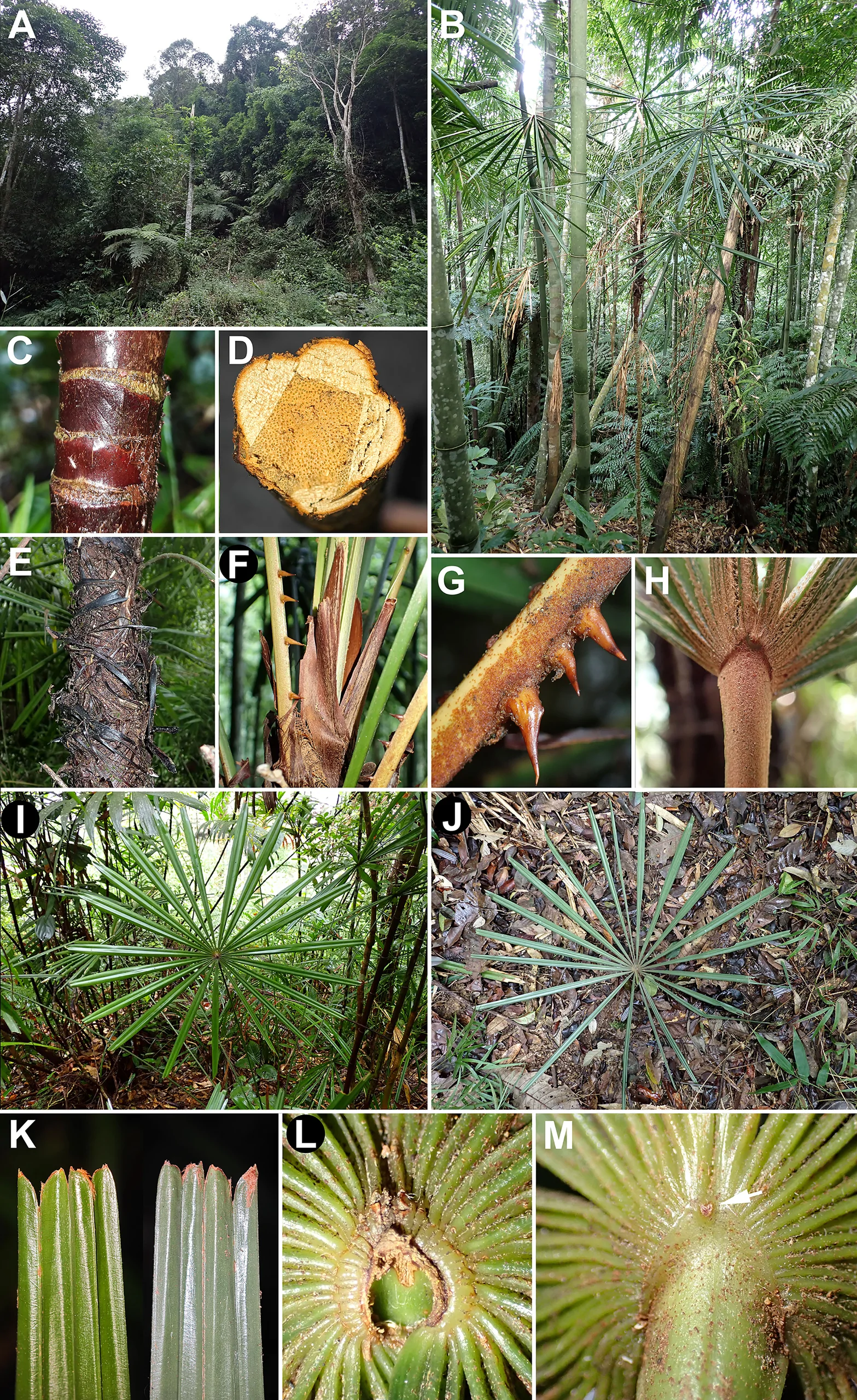
*Lanonia nhatii* sp. nov., habitat and details of vegetative structures. **A**. Habitat; **B**. Mature plant; **C**. Basal portion of stem, showing the bare surface; **D**. Cross-section of stem; **E**. Apical portion of stem, covered by leaf sheath fibers; **F**. Ligules; **G**. Petiole thorns; **H**. Abaxial surface of new leaf, densely covered with ferruginous hairs; **I**. Adaxial surface of leaf; **J**. Abaxial surface of leaf; **K**. Apex of central segment, adaxial view (left) and abaxial view (right); **L**. Hastula; **M**. Costa, with the pulvinus structure at the apex indicated by the arrow. All photographs by Jia-Wei Qin.

**Figure 2.**
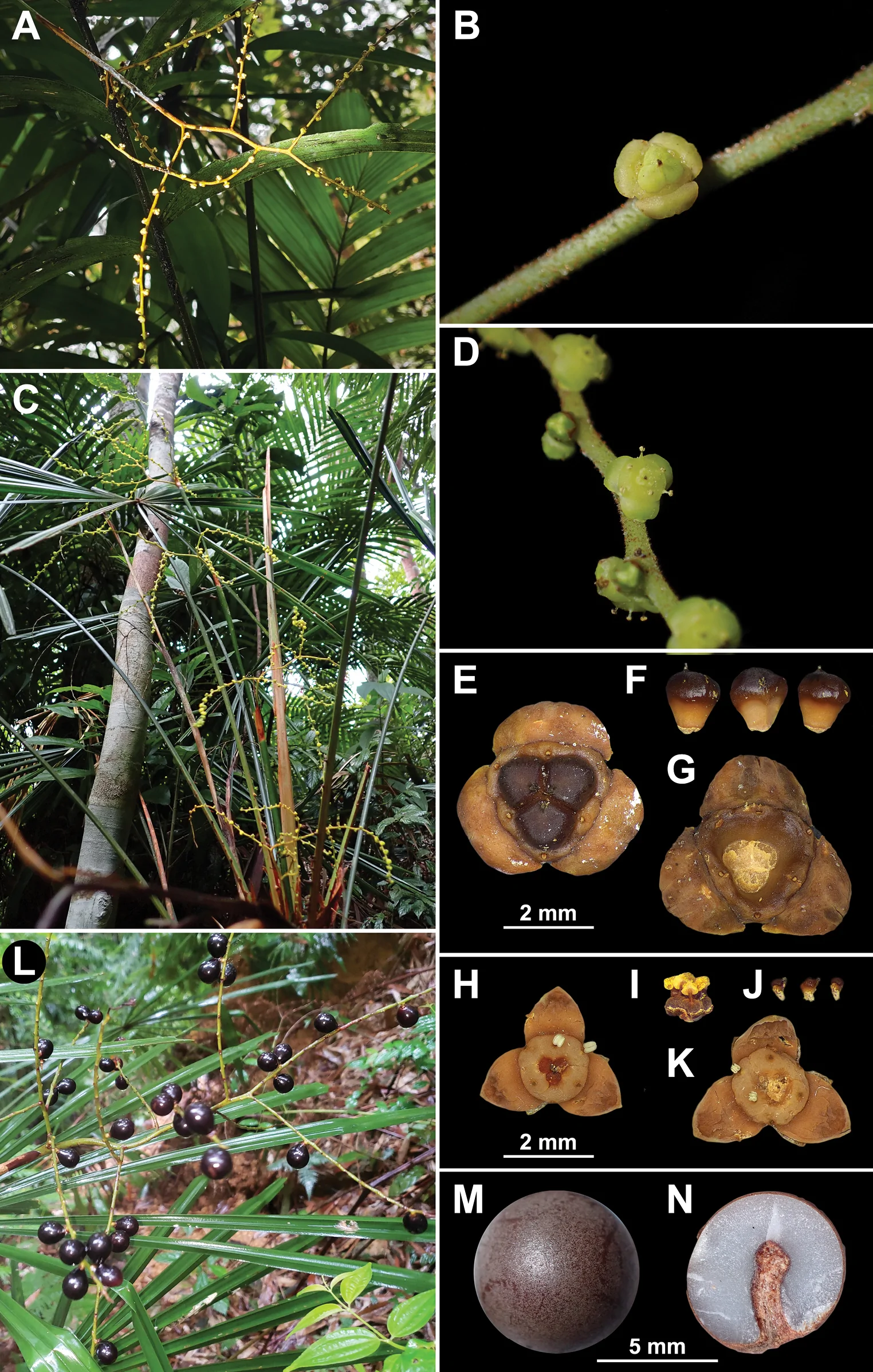
*Lanonia nhatii* sp. nov., details of reproductive structures. **A**. Pistillate inflorescence, showing a partial inflorescence; **B**. Pistillate flower solitary on the rachilla; **C**. Staminate inflorescence; **D**. Staminate flowers clustered two or three together on the rachilla; **E**. Pistillate flower specimen; **F**. Three apocarpous carpels of pistillate flower; **G**. Pistillate flower with the gynoecium removed, showing a staminodial ring; **H**. Staminate flower specimen; **I**. Staminal ring of staminate flower; **J**. Three apocarpous pistillodes of staminate flower; **K**. Staminate flower with the pistillodes removed, showing a staminal ring; **L**. Purplish-black mature fruits on infructescence; **M**. Seed surface; **N**. Longitudinal section of seed interior, with a straight but slightly asymmetrical basal intrusion. Scale bars: 5 mm (**M, N**); 2 mm (**E–K**). Photographs: **A–D**, Jia-Wei Qin; **E–K**, Zhi-Ling Tian; **L**, Trieu Van Nhat; **M, N**, Zheng-Shun Chen.

##### Diagnosis.

The new species differs from all congeneric species in an extremely short costa (the main axis of the leaf blade, i.e., the petiole extension into the blade) and purplish-black mature fruits. Additionally, the slender central segments and the elongate inflorescences represent two rare morphological traits within the genus.

##### Description.

***Plant*** perennial, dioecious. ***Stem*** 130.8 (66.0–295.0) cm long, 3.1 (2.3–4.5) cm in diameter, solitary or clustered. ***Leaves*** 13 (9–17) per stem; ligules 9.5 (8.0–19.0) cm long, papery, not disintegrating into fibers; sheaths and proximalmost part of petioles with dense, brown scales; petioles 91.8 (62.0–125.0) cm long, 0.5 (0.2–0.7) cm wide at the apex; petiole thorns usually poorly developed, brown or black, more or less regularly arranged on proximalmost part of petiole; hastulas raised, triangular, infolded; leaf blades 42.2 (32.0–52.0) cm long, 72.5 (56.5–89.0) cm wide; costa 0.2 (0.1–0.3) cm long, triangular, with a pulvinus at the apex abaxially, with the numerous segments free to the base; segments 20 (17–21) per leaf, equal in size, not mottled, with reddish-brown scales abaxially; central segments 42.2 (32.0–52.0) cm long, 1.6 (1.1–2.5) cm wide at the apex, not wider than the lateral segments; apices of central segments with adaxial splits not much deeper than abaxial ones. ***Inflorescences*** interfoliar, sexually dimorphic, with staminate and pistillate inflorescences and their peduncles unequal in length; peduncular bracts absent in staminate inflorescence, 3 (2–4) in pistillate inflorescence; rachis bracts tubular, splitting apically, not densely tomentose; rachillae filiform, sparsely to moderately covered with short, mostly simple, brownish hairs; ***staminate*** inflorescence 110.8 (78.0–148.0) cm long, with 4–7 partial inflorescences, branched to 3–4 orders; prophyll 7.0 (6.0–9.0) cm long; peduncles 21.4 (6.0–63.0) cm long; rachises 70.9 (26.0–96.0) cm long; rachillae 67 (25–122), 9.6 (5.0–15.6) cm long, 0.2 (0.1–0.3) mm in diameter, green; flowers 2–3 per cluster, 1.0 (0.8–1.2) mm long, 2.6 (2.0–3.0) mm in diameter; calyx 3-lobed, greenish-yellow, sepals not pedicellate at the base, sparsely hairy; corolla comprising three triangular-orbicular petals, greenish-yellow, apex acute; stamens six, with short filaments fused proximally into a staminal ring, and small, almost square anthers; pistillodes three; ***pistillate*** inflorescence reduced to a single partial inflorescence, branched to 2–3 orders, 122.4 (90.0–190.0) cm long; prophyll 7.8 (2.5–11.6) cm long; peduncles 84.3 (57.0–130.0) cm long; rachises absent; rachillae 7 (4–12), 12.3 (8.5–16.6) cm long, 0.3 (0.1–0.5) mm in diameter, green; flowers solitary, 2.6 (2.0–3.5) mm long, 3.8 (3.0–4.0) mm in diameter; calyx 3-lobed, greenish-yellow, not densely hairy; corolla comprising three semicircular petals, greenish-yellow, apex rounded; vestigial stamens six, with short filaments fused proximally into a staminodial ring; gynoecium apocarpous, carpels three. ***Fruits*** size not recorded, globose, purplish-black at maturity, with smooth surfaces; ***Seeds*** 7.9 (7.1–8.9) mm in diameter, globose, with a straight but slightly asymmetrical basal intrusion.

##### Etymology.

The specific epithet is named in honor of Mr. Trieu Van Nhat, the first discoverer of this species.

##### Phenology.

Flowering January to February, fruiting October to November.

##### Distribution and habit.

*Lanonia nhatii* is only from Chieng Ken Commune, Lao Cai Province, Vietnam. It grows in primary evergreen broad-leaved forest and also occurs in bamboo forest, at elevations ranging from 550 m to 670 m a.s.l. (Fig. 1A, B).

##### Phylogenetic position.

**Figure 3.**
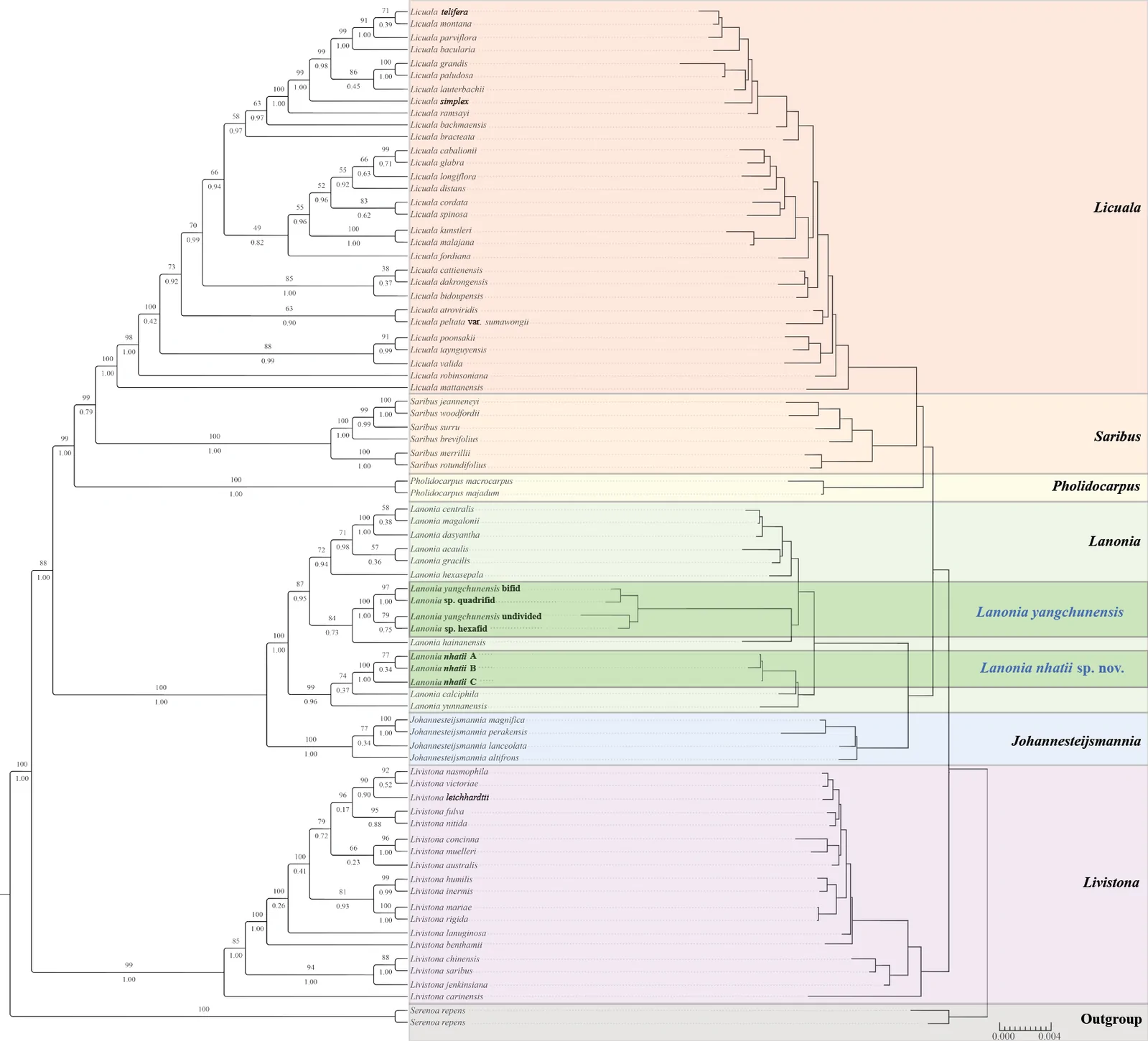
Phylogeny of *Lanonia nhatii* sp. nov. and *Lanonia yangchunensis* and their related species in Livistoninae, rooted using *Serenoa repens* as the outgroup. ML bootstrap percentages are shown above each branch, while BI posterior probabilities are shown below. The samples of the two focal species in this study are highlighted in bold font with a light green background.

Phylogenetic analyses placed *Lanonia nhatii* within *Lanonia* in a highly supported monophyletic clade (bootstrap percentage [BP] = 100, posterior probability [PP] = 1.00), where it is sister to *Lanonia calciphila* (Becc.) A.J.Hend. & C.D.Bacon, its closest relative (Fig. 3).

##### Taxonomic notes.

Apart from the two diagnostic features that distinguish *Lanonia nhatii* from all other species of the genus, namely a 0.1–0.3 cm long costa and purplish-black mature fruits, it is strikingly different from its closely related species *L. calciphila* and *L. yunnanensis* by the combination of leaves with numerous, approximately equal segments, papery ligules that do not disintegrate into fibers, a greater number of staminate partial inflorescences, and greenish-yellow sepals, petals, and rachillae.

#### Lanonia yangchunensis

Taxon classificationPlantaeArecalesArecaceae

Y.S.Chen, Phytotaxa 742: 86. 2026.

8F5F2171-0B44-57F9-8E69-683EACC65ACF

Figs 4, 5, 6

##### Type.

China • Guangdong Province, Yangchun City, Ehuangzhang Provincial Nature Reserve, elevation ca. 400 m a. s. l., growing in understory, 28 February 2025, *Z.C. Jin*, *L. Li & B.Y. Zhang ZL2025013* (holotype: IBSC! [IBSC1083374]; isotypes: IBSC! [IBSC1083375]).

**Figure 4.**
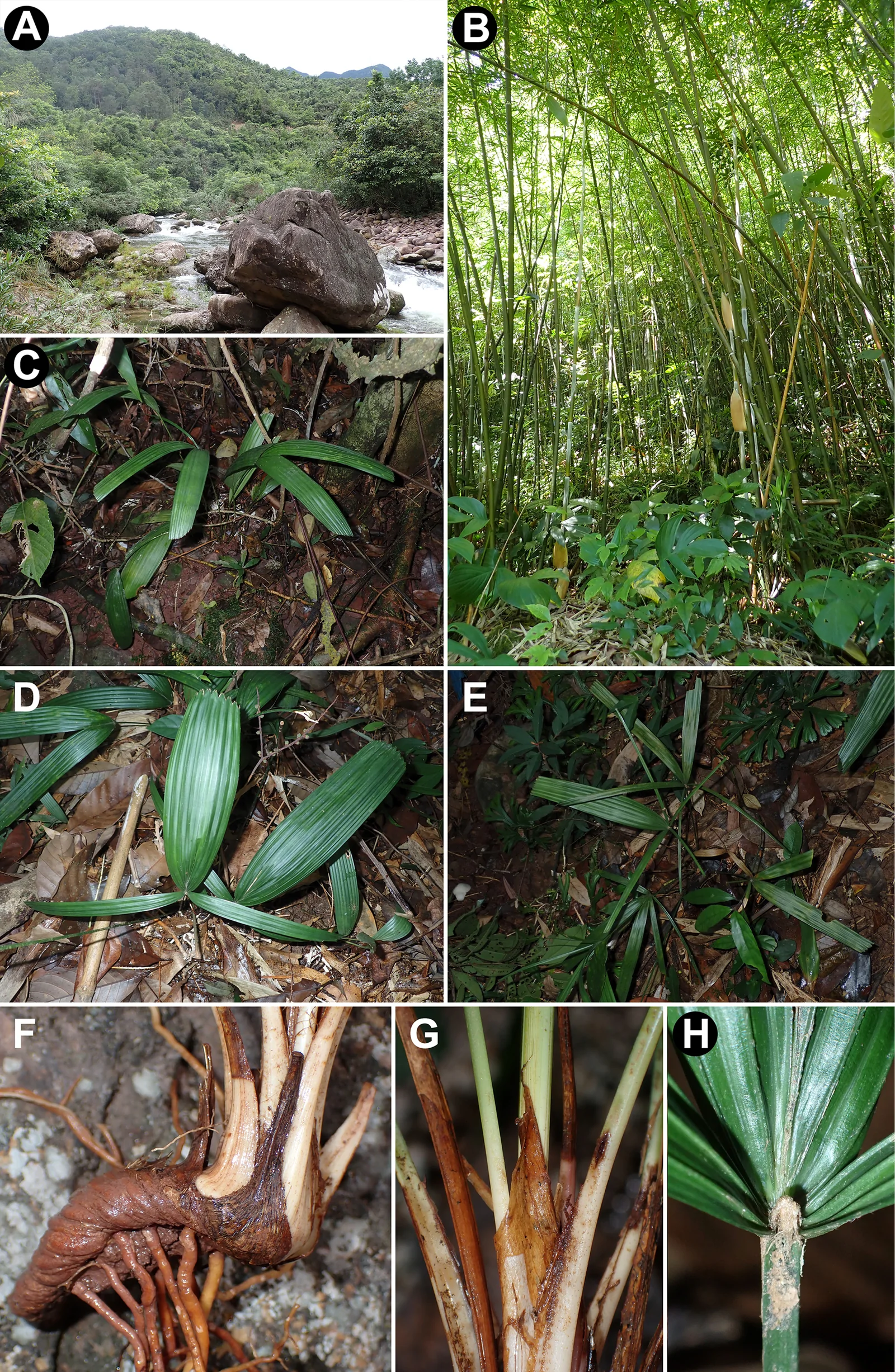
*Lanonia yangchunensis*, habitat and details of vegetative structures. **A, B**. Habitat; **C–E**. Mature plants; **F**. Stem, roots, and leaf sheaths; **G**. Ligules; **H**. Hastula. Photographs: **A–E**. Zhi-Ling Tian; **F–H**. Jia-Wei Qin.

**Figure 5.**
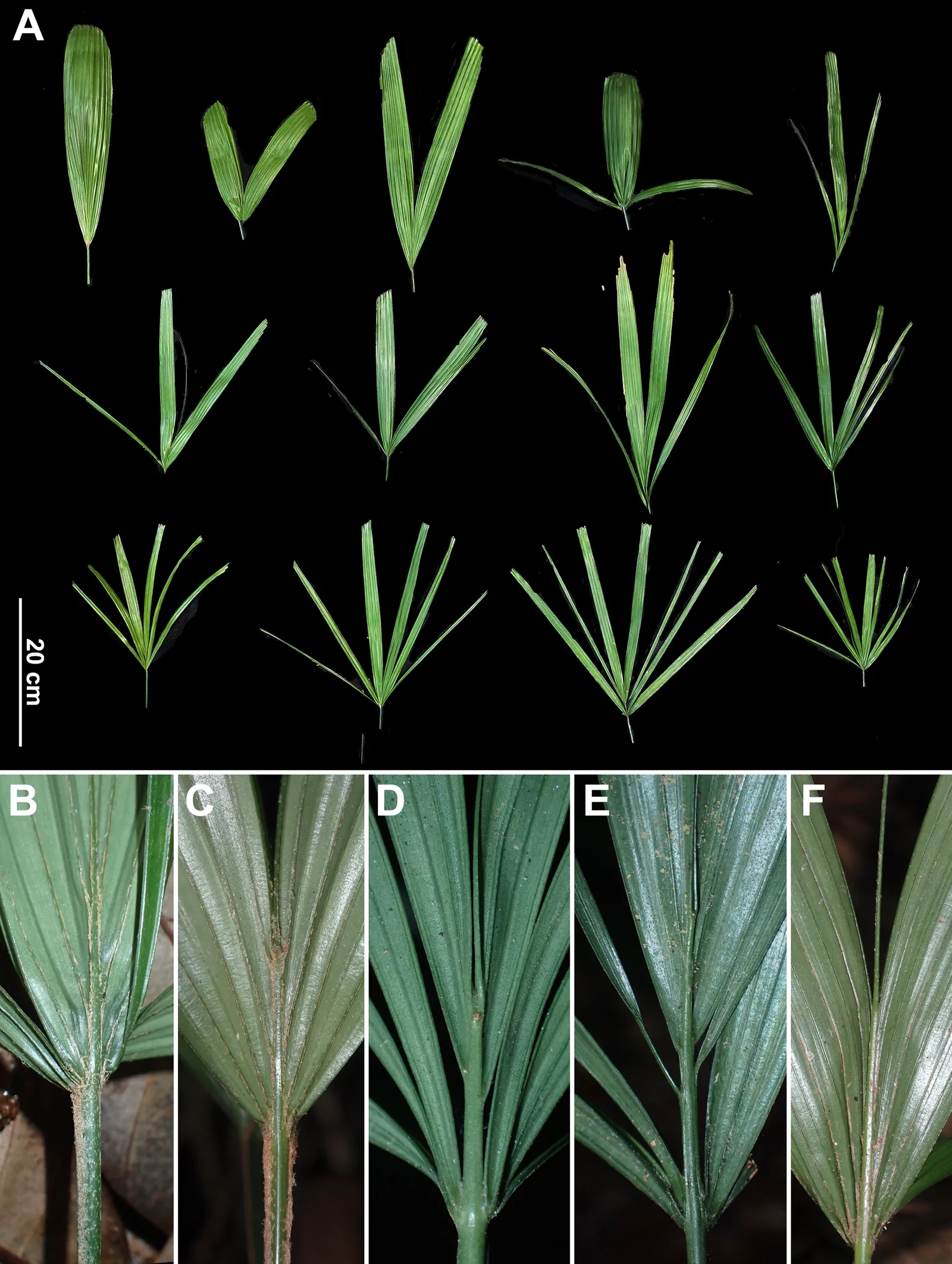
*Lanonia yangchunensis*, leaf morphotypes. **A**. Different segmentation types in mature plants; **B**. Central segment undivided and costa continuous to the leaf apex, without an abaxial pulvinus; **C**. Central segment divided but without a petiolule, and costa not continuous, with a pulvinus at the apex abaxially; **D**. Central segment divided with a petiolule, and costa not continuous, with a pulvinus at the apex abaxially; **E**. Central segment divided with a petiolule, pinnate, and costa not continuous, with a pulvinus at the apex abaxially; **F**. Central segment divided, but costa continuous to the leaf apex, without an abaxial pulvinus. Scale bar: 20 cm (**A**). Photographs: **A**. Zhi-Ling Tian; **B–F**. Jia-Wei Qin.

**Figure 6.**
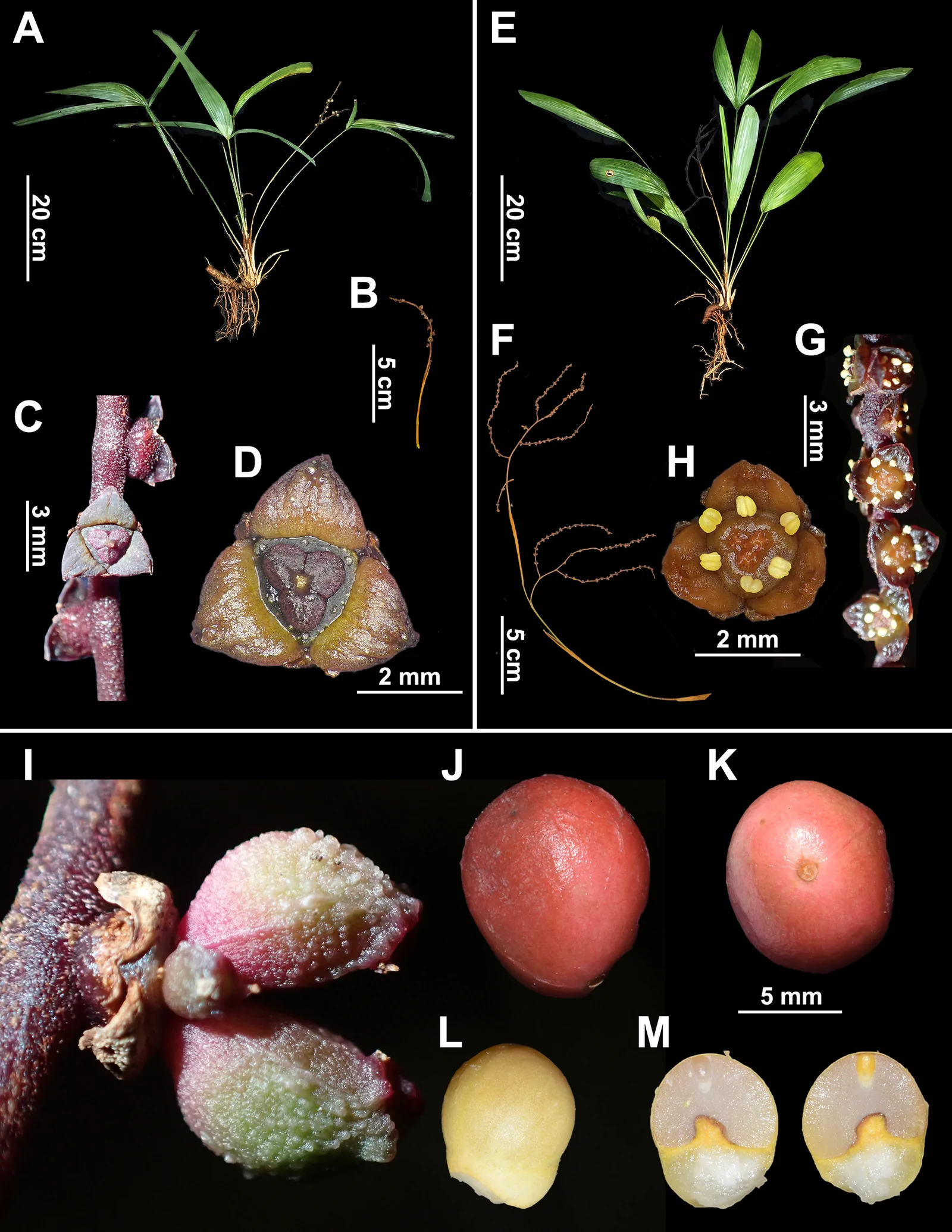
*Lanonia yangchunensis*, details of reproductive structures. **A**. Pistillate plant; **B**. Pistillate inflorescence; **C**. Pistillate flower solitary on the rachilla; **D**. Pistillate flower specimen; **E**. Staminate plant; **F**. Staminate inflorescence; **G**. Staminate flowers solitary or clustered in pairs on the rachilla; **H**. Staminate flower specimen; **I**. Three apocarpous carpels independently developing into three immature fruits with uneven surfaces; **J**. Fruit, lateral view; **K**. Fruit, view of the attachment surface to the rachilla; **L**. Seed surface; **M**. Longitudinal section of seed interior, with a short, straight but asymmetrical basal intrusion. Scale bars: 20 cm (**A, E**); 5 cm (**B, F**); 5 mm (**J–M**); 3 mm (**C, G**); 2 mm (**D, H**). Photographs: **A–G**. Zhi-Ling Tian; **I–M**. Zheng-Shun Chen.

##### Supplementary description‌.

***Plant*** perennial, dioecious. ***Stems*** subterranean, up to 9.0 cm long, elliptic in cross-section, 0.7–2.0 × 0.3–1.5 cm, solitary. ***Leaves*** 6 (3–8); ligules 8.0 (7.0–9.0) cm long, papery, not disintegrating into fibers; sheaths and proximalmost part of petioles with scattered, brown scales; petioles 32.4 (6.5–85.0) cm long, 0.2 (0.1–0.3) cm wide at the apex; petiole thorns absent; hastulas raised, triangular, infolded; leaf blades 59.7 (18.0–85.0) cm long, 33.0 (5.0–54.0) cm wide; costa 3.5 (0.4–11.0) cm long, relatively short or long and narrow, with a pulvinus at the apex abaxially, with the few segments free and the central pair free to the base or joined at their bases or borne on a 0.7 (0.3–1.0) cm long petiolule, or 28.8 (17.0–51.0) cm long, continuous to apex of leaf, without an abaxial pulvinus; segments variable in number among individuals, from undivided to variously divided up to nine segments, not mottled, with minute, reddish-brown scales abaxially; central segments undivided to variously divided into bifid segments to pinnately segmented of up to five segments on each side, 20.9 (10.0–33.5) cm long, 1.8 (0.4–3.2) cm wide at the apex in split ones, 30.8 (17.0–52.0) cm long, 3.7 (2.0–5.0) cm wide at the apex in undivided ones, wider or not wider than the lateral segments; apices of central segments with adaxial splits not much deeper than abaxial ones. ***Inflorescences*** interfoliar, sexually dimorphic, with staminate and pistillate inflorescences conspicuously differing in rachilla number; peduncular bracts absent, rarely one in pistillate inflorescence; rachis bracts narrowly tubular, splitting apically, sparsely tomentose; rachillae filiform, densely covered with lepidote, reddish-brown hairs; ***staminate*** inflorescence reduced to a single partial inflorescence or with two partial inflorescences, branched to 2–4 orders, 38.8 (23.7–59.0) cm long; prophyll 9.5 (4.6–17.0) cm long; peduncle 10.5 (7.0–16.0) cm long; rachises absent or 18.0 (10.0–24.0) cm long; rachillae 8 (3–12), 7.7 (5.5–10.5) cm long, 0.1 mm in diameter, purple; flowers solitary or two per cluster, 1.5 (1.0–1.8) mm long, ca. 2.9 mm in diameter; calyx 3-lobed, dark purple, sepals not pedicellate at the base, densely hairy; corolla comprising three triangular petals, dark purple, apex acute; stamens six, with short filaments fused proximally into a staminal ring, and small, almost square anthers; pistillodes three; ***pistillate*** inflorescence reduced to a single partial inflorescence, rarely two, branched to 1–2 orders, 30.7 (10.0–44.0) cm long; prophyll 7.3 (2.7–12.0) cm long; peduncles 9.6 (3.7–15.0) cm long; rachises usually absent, rarely 10.5 (7.5–13.5) cm long; rachillae 2 (1–3), 7.5 (4.3–10.8) cm long, 0.1 mm in diameter, purple; flowers solitary, 2.4 (2.0–2.6) mm long, ca. 3.9 mm in diameter; calyx 3-lobed, dark purple, densely hairy; corolla comprising three triangular petals, dark purple, apex acute; vestigial stamens six, with short filaments fused proximally into a staminodial ring; gynoecium apocarpous, carpels three. ***Fruits*** 12.0 (9. 0–13.0) mm long, 10.5 (8.0–11.0) cm wide, ellipsoid, red at maturity, with uneven surfaces in immature stage and smooth surfaces at full maturity; ***Seeds*** 8.5 (8.0–9.0) mm in diameter, globose, with a short, straight but asymmetrical basal intrusion.

##### Distribution and habitat.

*Lanonia yangchunensis* was first discovered in Ehuangzhang Provincial Nature Reserve, Yangchun, Guangdong Province, China. In this study, it was found near the Sino-Vietnamese border in southwestern Guangxi Zhuang Autonomous Region, China, ca. 400 km from the type locality. The populations in Guangxi have a wider distribution range, occurring in the understory of montane evergreen broad-leaved forests and bamboo forests at elevations of 50–900 m a.s.l. (Fig. 4A, B).

##### Additional specimens examined.

China • Guangxi Zhuang Autonomous Region, Fangchenggang City, precise locality withheld, elevation 50 m a.s.l., growing in the understory of montane evergreen broad-leaved forest, 29 April 2026, *Jia-Wei Qin & Zhi-Ling Tian, QJW-GXFC 260429* (HNNU!); • ibid., elevation 900 m a.s.l., 30 April 2026, *Jia-Wei Qin & Zhi-Ling Tian, QJW-GXFC 260430* (HNNU!).

##### Taxonomic notes.

During field investigations along the Sino-Vietnamese border in 2024, a peculiar palm population was discovered. It could not initially be confirmed whether it represented one or multiple distinct species because of its remarkably wide variation in leaf morphology. After careful comparison of floral characters, the individuals with undivided and bifid leaves were verified to correspond to the species newly published in early 2026, *L. yangchunensis*. Individuals with undivided, bifid, quadrifid, and hexafid leaf forms were subsequently sampled for phylogenetic analysis. The results showed that all individuals with different leaf forms clustered into a single monophyletic clade (BP = 100, PP = 1.00) (Fig. 3). Combined with comparative morphological observations of inflorescences, the results confirmed that all these leaf forms belong to *L. yangchunensis*.

Multiple leaf morphotypes co-occur within each population in Guangxi and can be divided into three groups: the central segment undivided (including the undivided leaf form) (Fig. 5B), the central segment divided but without a petiolule (including the bifid leaf form) (Fig. 5C), and the central segment divided with a petiolule (Fig. 5D). One individual with a pinnate central segment (Fig. 5E) and another with a divided central segment but a continuous costa (Fig. 5F) were found to be exceptions. Furthermore, some mature individuals consistently produce asymmetric leaves, with unequal numbers of lateral segments on the two sides of the central segment.

Thus, the supplementary description is provided based on individuals from wild populations in Guangxi, China. All measurements were taken from mature wild individuals, excluding juvenile plants. This description supplements the range of morphological variation and newly recorded stable characters that were not covered in the original protologue based on a single population from Guangdong, China.

### Pollen grains

**Figure 7.**
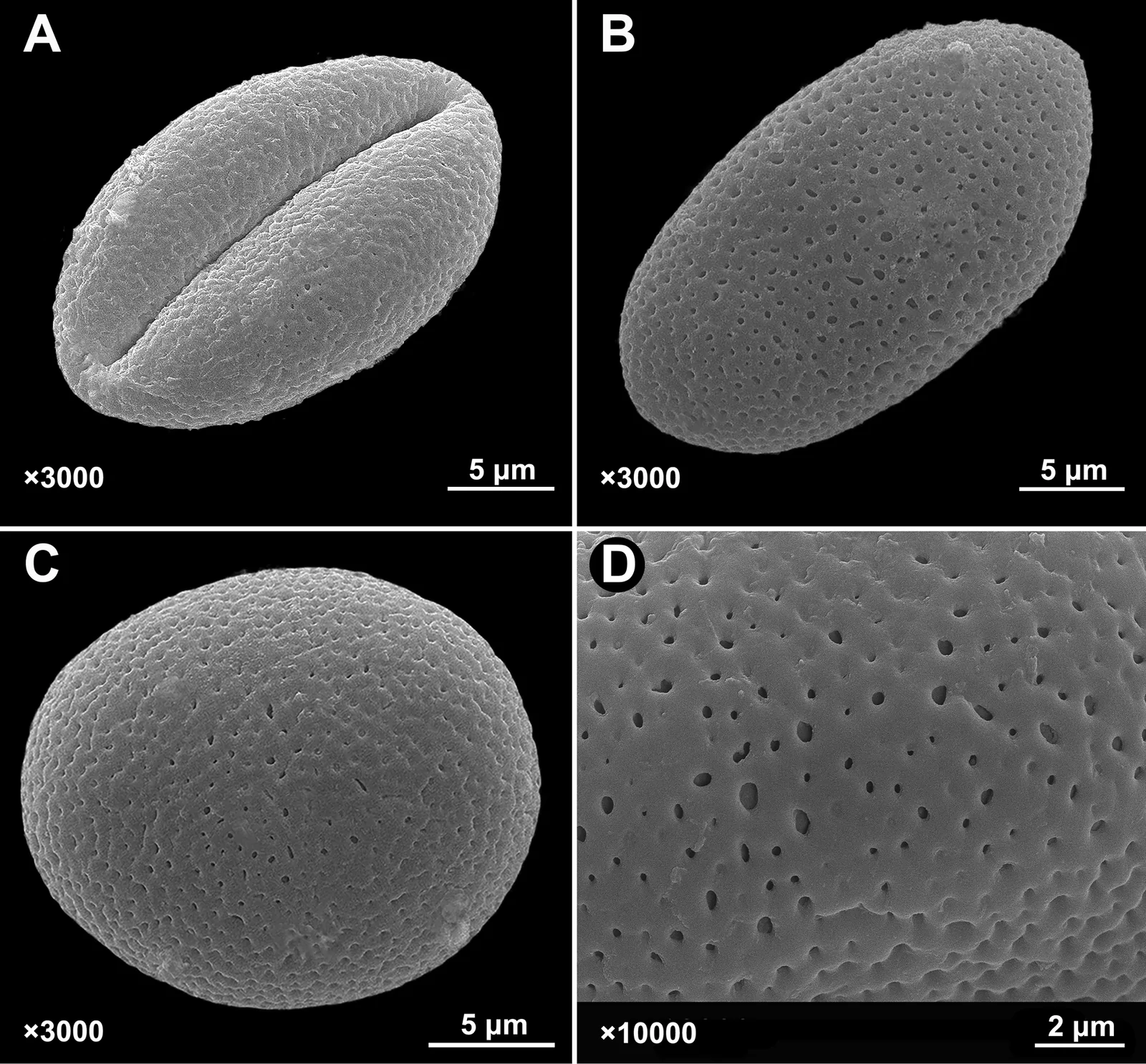
SEM photographs of pollen grains of *Lanonia nhatii* sp. nov. **A, B**. Equatorial view; **C**. Polar view; **D**. Exine ornamentation. Scale bars: 5 μm (**A–C**); 1 μm (**D**). Photographs: Zhi-Ling Tian.

Pollen grains of *L. nhatii* (Fig. 7) are monads, monocolpate, and long-elliptical in shape, long-elliptical in equatorial view, and elliptical in polar view. The polar axis is 25.61 (23.20–28.28) μm long, and the equatorial axis is 16.11 (14.19–18.19) μm long, with a *P/E* ratio of 1.60. The exine is tectate, with foveolate ornamentation. The lumina are circular in shape and variable in size, 0.26 (0.13–0.45) μm in diameter.

**Figure 8.**
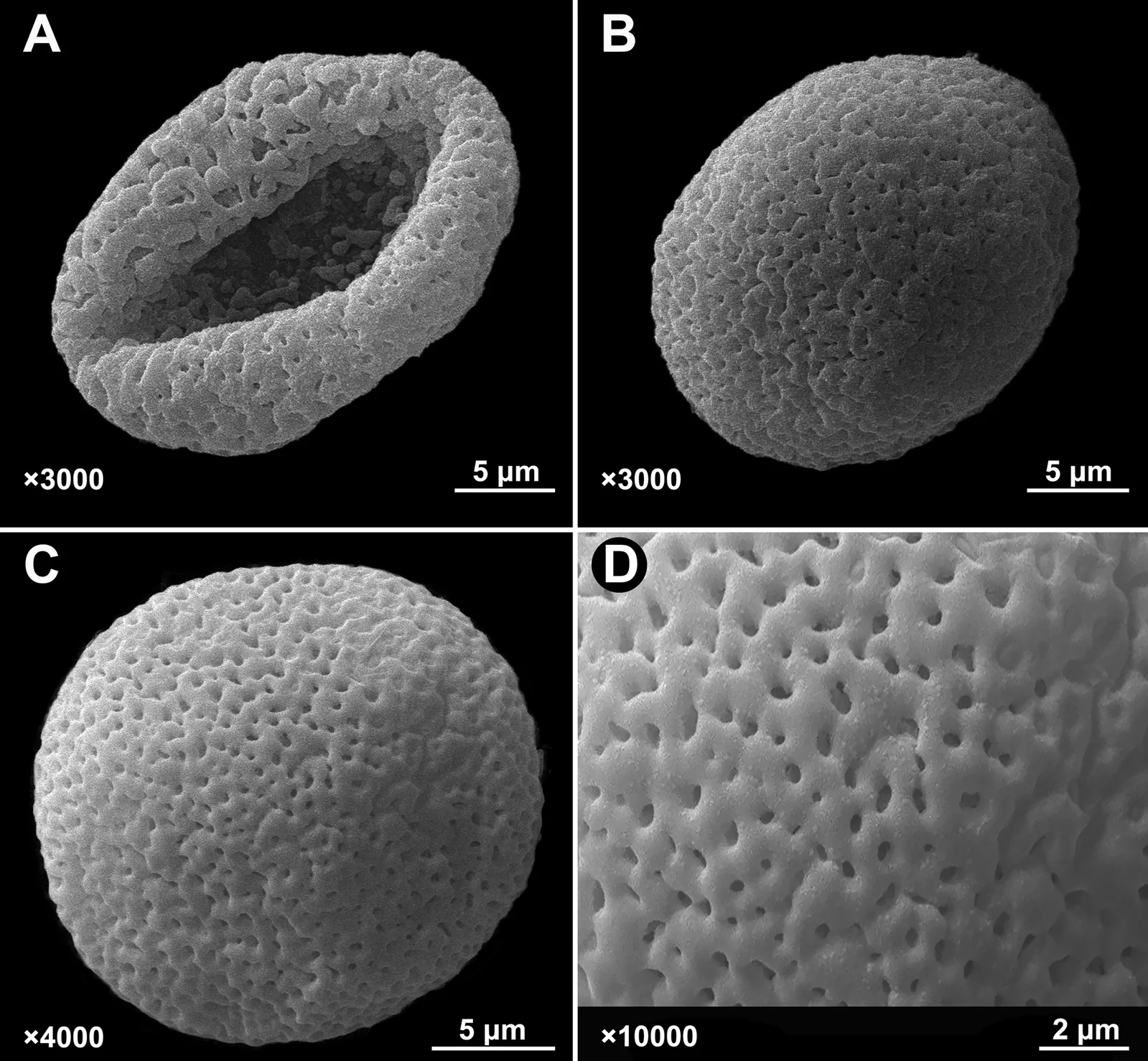
SEM photographs of pollen grains of *Lanonia yangchunensis*. **A, B**. Equatorial view; **C**. Polar view; **D**. Exine ornamentation. Scale bars: 5 μm (**A–C**); 1 μm (**D**). Photographs: Zhi-Ling Tian.

Pollen grains of *L. yangchunensis* (Fig. 8) are monads, monocolpate, elliptical in equatorial view, and nearly round in polar view. The polar axis is 21.34 (18.48–23.48) μm long, and the equatorial axis is 16.01 (13.15–17.69) μm long, with a *P/E* ratio of 1.34. The exine is tectate with reticulate ornamentation; both the lumina and muri bear microperforations. The lumina are irregular in shape, 0.40 (0.18–0.62) μm in diameter, while the muri are 0.53 (0.23–0.84) μm wide and granulate on the surface.

## Discussion

The sexual dimorphism of *L. nhatii* sometimes shows ambiguity. In some male individuals, the pistillode was found to expand significantly after anthesis and develop into an immature fruit. Furthermore, one inflorescence specimen was found to exhibit a branching pattern more similar to that of male inflorescences in the number of partial inflorescences and rachillae, while its inflorescence length was closer to that of female inflorescences, and immature fruits developed on every partial inflorescence.

Palm pollen is generally conserved (Dransfield et al. 2008), and characters such as size, shape, exine ornamentation, and aperture type can provide supplementary evidence for the identification of some species (Rahim et al. 2025). The pollen morphology of the subfamily Coryphoideae is relatively consistent, and pollen of the former tribe Corypheae is typically monosulcate and elliptic to subcircular in polar view (Dransfield et al. 1990). Although there is a paucity of specialized research on the pollen morphology of *Lanonia*, that of its related genus, *Licuala*, was reported by Ambwani and Kumar (1993). Based on the differences in exine ornamentation observed among nine species of the genus *Licuala*, Ambwani and Kumar (1993) classified the pollen of this genus into four types: coarsely reticulate, microreticulate, finely reticulate, and perforate. The pollen grains of *L. nhatii* possess a foveolate exine, which distinguishes them from all four pollen types of *Licuala* summarized by Ambwani and Kumar (1993). However, Dransfield et al. (2008) documented that both *Licuala* and *Livistona* in Livistoninae have a foveolate ectexine. The exine of pollen grains of *L. yangchunensis* has both reticulate and perforate ornamentation, a character differing from those of the four types described by Ambwani and Kumar (1993). Dransfield et al. (1990) stated that *Licuala* exhibits great variation in tectal structure, ranging from finely and sparsely perforate to reticulate. The reticulate-perforate exine tectum of *L. yangchunensis* is likely an intermediate form. Pollen morphological differences were observed between the two species examined in this study. *L. nhatii* possesses larger pollen grains but smaller lumina, resulting in a higher lumen density per unit area, and under SEM, it has a relatively smooth surface with a linear colpus, whereas *L. yangchunensis* shows the reverse condition, with smaller pollen grains but larger lumina and lower lumen density per unit area, and it exhibits densely distributed granular processes on the muri and has a wider colpus. No shared pollen characters representative of the genus *Lanonia* have yet been identified for *L. nhatii* and *L. yangchunensis*. Further study is needed to determine whether exine sculpture in *Lanonia* is a taxonomically valuable character for species identification and whether it can serve as a distinguishing feature from *Licuala*.

## Supplementary Material

XML Treatment for Lanonia nhatii

XML Treatment for Lanonia yangchunensis

## Additional information

### Artificial Intelligence (AI) use

The authors accept full responsibility for the content of the manuscript, including the disclosure of any use of AI.

No AI tools were used in the preparation of this manuscript.

### Author ORCIDs

J.-W. Qin https://orcid.org/0009-0008-3280-0076

Z.-L. Tian https://orcid.org/0009-0003-7772-0866

Z.-S. Chen https://orcid.org/0009-0007-8959-0789

K.S. Nguyen https://orcid.org/0000-0001-5171-4140

R.-P. Kuang https://orcid.org/0009-0001-9743-9129

### Data availability

All of the data that support the findings of this study are available in the main text or Supplementary Information.

## Supplementary materials

Supplementary material 1 Genbank accession numbers Data type xlsx Explanation note The accession numbers of all nucleotide sequences used in this study (both novel sequences and those derived from GenBank) are listed in Suppl. material 1. https://binary.pensoft.net/file/1777497This dataset is made available under the Open Database License (http://opendatacommons.org/licenses/odbl/1.0/). The Open Database License (ODbL) is a license agreement intended to allow users to freely share, modify, and use this Dataset while maintaining this same freedom for others, provided that the original source and author(s) are credited.Author: Jia-Wei Qin
